# Prevalence and Molecular Characterization of *Enterocytozoon bieneusi* in Horses From Northern and Eastern China: Investigation of Associated Risk Factors and Assessment of Zoonotic Potential

**DOI:** 10.1155/tbed/1144348

**Published:** 2026-08-31

**Authors:** Xin Peng, Zhongkai Zhang, Chuhan Yan, Fanggu Zhu, Weimeng Qin, Wei Zhao, Jiangqiong Ke

**Affiliations:** ^1^ Department of Geriatrics, The Second Affiliated Hospital and Yuying Children’s Hospital of Wenzhou Medical University, Wenzhou 325027, Zhejiang, China, wmu.edu.cn; ^2^ The First Clinical Medical College of Wenzhou Medical University, Wenzhou 325035, Zhejiang, China; ^3^ School of Basic Medical Sciences, Wenzhou Medical University, Wenzhou 325035, Zhejiang, China, wmu.edu.cn

**Keywords:** *Enterocytozoon bieneusi*, horse, ITS genotype China, molecular epidemiology, One Health, zoonosis

## Abstract

*Enterocytozoon bieneusi* is a zoonotic microsporidian threatening public health, yet epidemiological data on equine infections in China are scarce despite frequent human–horse contact. In this study, 412 fresh horse fecal samples were collected from four Chinese regions covering four rearing systems and were screened for *E. bieneusi* internal transcribed spacer (ITS) sequences via nested PCR, with risk factor and phylogenetic analyses conducted. The overall infection prevalence reached 15.0% (62/412), varying geographically from 9.7% (Wenzhou) to 29.3% (Qiqihar). Foals <1 year and male horses exhibited significantly higher infection risks, with crude prevalence ratios (PRs) of 4.47 and 2.28, respectively, while prevalence did not differ statistically across rearing modes (*p* = 0.182). Eighteen genotypes were identified: 11 known (D, horse1, Peru8, Type IV, EbpC, CAF1, GX3, HNR‐VI, SHWR5, SYSWR18, SYSWR23) and seven novel (HRBM1, KDM1, KDM2, QQHRM1, QQHRM2, QQHRM3, WZM1). Genotype D was dominant, accounting for 58.1% (36/62) of positive isolates, followed by genotype horse1 at 14.5% (9/62) and genotype EbpC at 3.2% (2/62). The other eight known genotypes and all seven novel genotypes each had one isolate. All seven novel genotypes clustered within zoonotic Group 1 with all the known genotypes except HNR‐VI identified here. This study confirms widespread equine *E. bieneusi* circulation across eastern and northern China, modulated by geography, age, and sex. The predominance of zoonotic genotypes implies horses serve as potential reservoirs for human transmission, supplying baseline epidemiological evidence for equine One Health biosecurity management.

## 1. Introduction

Microsporidia constitute a monophyletic phylum within the fungal kingdom, comprising over 220 genera and more than 1400 obligate intracellular species, at least 17 of which have been documented to humans [1]. *Enterocytozoon bieneusi* is the most prevalent microsporidian species infecting humans, accounting for more than 90% of all diagnosed microsporidiosis cases globally [2]. Infection occurs primarily via the fecal–oral route through ingestion of water or food contaminated with infectious spores shed in host feces [3]. Its chitin‐rich spore wall confers strong resistance to conventional chlorination, ultraviolet irradiation, and common agricultural disinfectants, allowing spores to persist for extended periods in soil, surface water, and sewage. This environmental stability sustains ongoing cross‐species transmission cycles [2].

*Enterocytozoon bieneusi* exhibits high genetic diversity. To date, more than 900 distinct genotypes have been identified in at least 210 animal species across more than 50 countries [4] and all defined through sequence analysis of the hypervariable internal transcribed spacer (ITS) region of the ribosomal RNA (*rRNA*) gene [5]. Phylogenetically, these genotypes cluster into 15 distinct groups with divergent host specificities and zoonotic potentials [6]. Group 1 is the most epidemiologically significant group, with the member genotypes capable of infecting a wide range of mammalian hosts. Over 90% of known zoonotic *E. bieneusi* genotypes belong to Group 1, supporting the hypothesis that members of this group have potential zoonotic transmission capacity [4]. Group 2 genotypes were historically considered ruminant‐adapted, but accumulating evidence has expanded their recognized host range and revealed moderate zoonotic potential. This is exemplified by genotypes BEB4, BEB6, I, and J, which have been detected in both humans and multiple animal species [3]. In contrast, Groups 3–15 consist mainly of host‐adapted genotypes with limited cross‐species transmissibility and low public health relevance [2]. As a result, different animal host species contribute unequally to the burden of human *E. bieneusi* infection. Species with frequent, close contact with humans carry a higher transmission risk and thus deserve prioritized epidemiological investigation.

Equines serve important roles in livestock production, cultural heritage, and equestrian sports worldwide and maintain frequent close contact with humans [7]. To date, *E. bieneusi* infection in equines has been reported in only eight countries, with prevalence ranging from 1.3% to 30.9%, and most regions have been covered by just a single published study (Table 1) [8–19]. Despite this limited body of research, at least 59 distinct *E. bieneusi* genotypes have been detected in horses, 14 of which (BEB6, CAF1, CHG19, CS‐4, CZ3, D, EbpA, EbpC, G, O, Peru6, Peru8, Type IV, and WL15) are widely recognized as having zoonotic potential (Table 1) [8–19]. This finding suggests that horses may function as environmental reservoirs for zoonotic *E. bieneusi* transmission to humans. Taken together, these observations underscore the need for larger‐scale epidemiological studies to clarify the zoonotic risk and transmission dynamics of *E. bieneusi* in horse populations across different regions and husbandry systems.

**Table 1:** Prevalence and genotype distribution of *E. bieneusi* infection in horses worldwide.

Country	No. positive/no. examined (%)	Genotype (no.)	% zoonotic genotypes	References
Algeria^a^	15/219 (6.9)	horse1 (6); horse2 (1); **CZ3 (2); D (1)**	20.0	[8]
Colombia	21/325 (6.5)	horse1 (13); horse2 (4); **D (4)**	19.0	[9]
Czech Republic	66/377 (17.5)	**D (34);** horse1 (7); horse2 (8); **G (3);** horse3 (2); horse11 (2); **WL15 (1)**; **EbpA (1)**; horse4–horse10 (each 1)	59.1	[10]
China	24/325 (7.4)	**J (7)**; horse1 (5); NMGH4 (2); NMGH5 (2); XJH5 (2); horse2 (1); **BEB6 (1)**; NMGH1–NMGH3 (each 1); XJH6 (1)	33.3	[11]
China	81/262 (30.9)	**BEB6 (9), CHG19 (2)**, **CM6 (4)**, CM7 (2), CM8 (1), CS‐1(1), **CS-4(1)**, **D (1)**, **EpbA (20), EbpC (21)**, **G (3)**, horse1 (4), horse2 (2), **O (4), Peru8 (1)**, XJH1 (2), XJH2 (1), XJH3 (1), XJH4 (1)	81.5	[12]
China	75/333 (22.5)	horse2 (39); SC02 (16); horse1 (13); **D (1)**; SCH1 (1); SCH2 (1); SCH3 (1); SCH4 (1); YNH1 (1); YNH2 (1)	1.3	[13]
China	5/32 (15.6)	CAM2 (5)	0.0	[14]
China	30/621 (4.8)	horse1 (16), **Peru6 (4), EbpC (2)**, horse2 (2), **EbpA (1), CAF1 (1), TypeIV (1)**, BJH‐1 (1), HBH‐1 (1), SXH‐1 (1)	30.0	[15]
Iran	3/26 (11.5)	**BEB6 (1)**; horse1 (1)	33.3	[16]
Republic of Korea	15/1200 (1.3)	horse1 (13); horse2 (2)	0.0	[17]
Turkey	56/300 (18.7)	ERUSS1 (24), **BEB6 (8)**, ERUH2 (6), ERUH3 (5), ERUH4 (4), ERUH5 (4), ERUH6 (3), ERUH7 (2)	14.3	[18]
United States	7/84 (8.3)	horse1 (7)	0.0	[19]

*Note:* The genotypes in bold font indicate that they can infect both humans and animals. Percentages in the “% zoonotic genotypes” column represent the proportion of positive isolates that belong to confirmed zoonotic *E. bieneusi* genotypes, not the proportion of zoonotic genotype variants among all detected genotypes.

^a^In the study conducted in Algeria, re‐sequencing of the five isolates was unsuccessful.

In China, however, molecular epidemiological data on equine *E. bieneusi* have not kept pace with the country’s fast‐growing equine industry. China’s equine sector exhibits geographically diverse husbandry practices. In northern pastoral regions, including Inner Mongolia, Heilongjiang, and Xinjiang, native breeds such as Mongolian horses and Heilongjiang horses are predominantly reared, primarily for meat production, labor use, and free‐range breeding, with frequent cross‐provincial live animal trade. In eastern coastal regions such as Zhejiang Province, imported warmblood breeds and thoroughbreds are mainly raised in intensive equestrian clubs and breeding farms, serving competitive equestrian sports, recreational riding, and tourism [20]. Horseback riding has surged in popularity among tourists across regions in recent years, increasingly serving as a hallmark of local tourist destinations. To date, only five epidemiological surveys of equine *E. bieneusi* have been conducted in China [11–15]. Most are limited to single provinces or single husbandry systems, and no study has systematically compared infection patterns across grazing, intensive farming, equestrian club, and live animal market settings. To address these knowledge gaps, we conducted a cross‐sectional molecular epidemiological survey of *E. bieneusi* in equines across four equine‐rearing regions in northern and eastern China: Heilongjiang, Inner Mongolia, Liaoning, and Zhejiang. This study provides baseline epidemiological data on equine *E. bieneusi* in China, advances understanding of the zoonotic risk posed by equine‐derived isolates, and provides evidence to support One Health biosecurity strategies for equine facilities and livestock trading markets.

## 2. Materials and Methods

### 2.1. Ethics Statement

The research protocol was reviewed and approved by the Research Ethics Committee of Wenzhou Medical University (Approval No. SCILL2021‐01). Prior to sample collection, verbal consent was obtained from animal owners whenever feasible. Only freshly voided fecal samples were collected, and no animal restraint or invasive procedures were performed during the sampling.

### 2.2. Study Sites and Sample Collection

**Figure 1:**
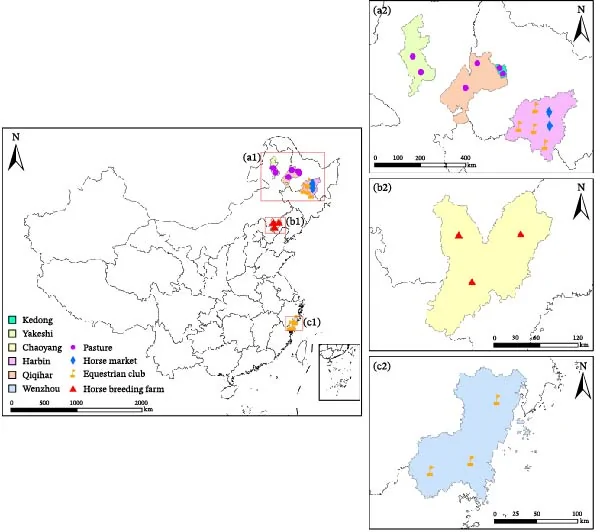
Geographic distribution of sampling sites for horses *E. bieneusi* surveillance in northern and eastern China. (a1, a2) Overview of sampling locations in Qiqihar, Kedong, and Harbin, Heilongjiang Province, and Yakeshi, Inner Mongolia Autonomous Region. (b1, b2) Distribution of sampling sites in Chaoyang, Liaoning Province. (c1, c2) Distribution of sampling sites in Wenzhou, Zhejiang Province. Different colored regions correspond to prefecture‐level sampling areas, and different symbols represent distinct husbandry systems and live animal trading markets.

A total of 412 fresh equine fecal samples were collected between October 2022 and October 2025. Samples were obtained from six sampling sites across four provincial‐level administrative regions in China. These regions included Heilongjiang Province (Harbin, *n* = 142; Kedong, *n* = 46; Qiqihar, *n* = 41), Inner Mongolia Autonomous Region (Yakeshi, *n* = 42), Liaoning Province (Chaoyang, *n* = 69), and Zhejiang Province (Wenzhou, *n* = 72). Sampling covered three typical equine husbandry systems: intensive breeding farms, free‐range pastures, and equestrian clubs. Live animal trading markets were included as a distinct epidemiological transmission setting. Overall, 18 independent equine establishments were enrolled in this study (Figure 1). Sampling coverage exceeded 90% of the resident horse population at each facility to minimize selection bias. Demographic information, including age, sex, and castration status, was recorded for each sampled horse. The study population was stratified into three age groups: <1 year (*n* = 29), 1–6 years (*n* = 115), and >6 years (*n* = 268). By sex, 120 horses were male (including 36 geldings), and 292 were female. Samples were grouped by husbandry type: 69 from intensive farms, 129 from free‐range grazing pastures, 147 from equestrian clubs, and 67 from two live animal trading markets. Approximately 30–50 g of fresh feces was collected from each horse immediately after spontaneous defecation. Each sample was sealed in a pre‐labeled sterile sampling bag. All samples were transported to the laboratory under ice‐cold conditions within 24 h of the collection. Samples were stored at 4°C until processing. DNA extraction was performed within 48 h of sampling to avoid nucleic acid degradation. Each horse was sampled only once to ensure statistical independence of all data points. None of the enrolled horses showed diarrhea or acute clinical symptoms at the time of sampling.

### 2.3. DNA Extraction

Fecal samples were sieved through a 250‐μm mesh to remove coarse debris, and the filtrate was centrifuged at 1500 × *g* for 10 min to pellet and concentrate spores. Genomic DNA was extracted from 200 mg of the resulting sediment using the QIAamp Fast DNA Stool Mini Kit (Qiagen, Hilden, Germany) following the manufacturer’s standard protocol. The concentration and purity of the extracted genomic DNA were determined spectrophotometrically using a NanoDrop 2000 spectrophotometer (Thermo Fisher Scientific, Waltham, MA, USA). Samples with an A260/A280 ratio of 1.8–2.0, indicating high‐purity DNA with minimal protein contamination, were selected for downstream molecular detection and stored at −20°C until further analysis.

### 2.4. Nested PCR Amplification of the ITS Region

Nested PCR was performed to amplify an ~390 bp fragment covering the entire 243 bp ITS region of the *E. bieneusi rRNA* gene using two validated primer pairs as previously described [21]. The outer primer pair EBITS3 (5^′^‐GGTCATAGGGATGAAGAG‐3^′^) and EBITS4 (5^′^‐TTCGAGTTCTTTCGCGCTC‐3^′^) were used for the primary amplification, while the inner primer pair EBITS1 (5^′^‐GCTCTGAATATCTATGGCT‐3^′^) and EBITS2.4 (5^′^‐ATCGCCGACGGATCCAAGTG‐3^′^) were used for the secondary nested reaction. Each 25 μL reaction mixture contained 2.5 μL of 10× PCR buffer, 2 μL of dNTP mix (2.5 mM each nucleotide), 0.5 μL of each forward and reverse primer (10 μM), 0.5 μL of Taq DNA polymerase (5 U/μL; TaKaRa), 2 μL of the genomic DNA template, and nuclease‐free water to bring to the final volume. For the nested round, 2 μL of the first‐round amplification product was used as the template. Genomic DNA of *E. bieneusi* genotype Peru11 [22] and double‐distilled water were included in every PCR run as positive and negative controls, respectively. The thermal cycling profile for the primary reaction consisted of an initial denaturation step at 94°C for 3 min, followed by 35 cycles of denaturation at 94°C for 30 s, annealing at 57°C for 30 s, and extension at 72°C for 40 s. The secondary nested reaction ran for 30 cycles with an adjusted annealing temperature of 55°C; denaturation and extension durations were identical to those of the primary assay. Amplified products were separated by 1.5% (w/v) agarose gel electrophoresis with GelRed nucleic acid staining and visualized under ultraviolet light alongside a 2000 bp DNA size marker. Samples showing a clear, specific band at the expected fragment size were scored as positive for *E. bieneusi*.

Strict contamination control protocols were implemented throughout the experimental workflow. Pre‐extraction sample handling, DNA extraction, PCR reaction setup, and post‐amplification product analysis were performed in four physically separated laboratory zones with dedicated pipettes and equipment. Aerosol‐resistant pipette tips were used for all liquid transfers to prevent amplicon carryover. All workbenches were UV‐irradiated for 30 min before and after each batch of experiments for surface decontamination.

### 2.5. Sequencing and Genotype Assignment

All positive amplicons of the expected size were purified and subjected to bidirectional Sanger sequencing by Sangon Biotech Co., Ltd. (Shanghai, China), using the inner nested PCR primers as sequencing primers. Raw sequencing chromatograms were first visually checked for signal quality, and low‐quality flanking regions were trimmed using Chromas v2.6.6 (Technelysium Pty Ltd., South Brisbane, Australia). Trimmed sequences were edited and assembled into high‐quality consensus sequences using Lasergene EditSeq v7.1 and the SeqMan module of the DNAStar software package (DNAStar Inc., Madison, WI, USA). Consensus sequences were queried against the NCBI GenBank database using BLASTn for preliminary species verification and genotype matching. Genotype assignment strictly followed the international consensus nomenclature criteria for *E. bieneusi* established by Santín and Fayer [5]. Sequences with 100% nucleotide identity to previously validated reference genotypes were classified as known genotypes. For isolates showing single or multiple nucleotide polymorphisms relative to known variants, duplicate independent nested PCR and bidirectional sequencing were repeated using the original genomic DNA template. Only nucleotide variations that were 100% concordant across two independent experimental runs were confirmed as authentic mutations, and the corresponding isolates were designated as novel genotypes. Novel genotypes identified in this study were named using the abbreviation of the sampling location and the first pinyin initials of the Chinese word for “horse” (Ma), followed by sequential Arabic numerals. For example, a novel genotype identified from a horse in Harbin was designated HRBM1. All representative genotype sequences obtained in this work have been deposited in the NCBI GenBank database under accession numbers PZ705401‐PZ705418.

### 2.6. Phylogenetic Analysis

A set of reference sequences representing all established *E. bieneusi* phylogenetic groups and key known genotypes was retrieved from NCBI GenBank for evolutionary analysis. Multiple sequence alignment was performed using the ClustalW algorithm implemented in MEGA 11 software (http://www.megasoftware.net/). The optimal nucleotide substitution model for phylogenetic reconstruction was selected based on the lowest Akaike information criterion (AIC) value calculated by the built‐in model selection function in MEGA 11. The Kimura 2‐parameter model with gamma‐distributed rate heterogeneity (K2+G) was identified as the best‐fit model for the ITS sequence dataset. A neighbor‐joining (NJ) phylogenetic tree was constructed under the selected substitution model. The reliability of the tree topology and nodal support was assessed using bootstrap resampling analysis with 1000 replicates. Bootstrap values of 50% or higher were displayed.

### 2.7. Statistical Analysis

Statistical analyses were performed using SPSS 22.0 (IBM, USA), with two‐sided *p*  < 0.05 set as the threshold for statistical significance. Prevalence and corresponding 95% Wilson score confidence intervals (CIs) were calculated overall and for each subgroup. Pearson’s chi‐square test was used when expected cell frequencies were ≥5, and Fisher’s exact test was applied when expected frequencies were <5. Due to the limited sample size of multiple age and regional subgroups, Fisher’s exact test was ultimately used for all prevalence comparisons in this study. Crude prevalence ratios (PRs) with 95% CIs were calculated via the Mantel–Haenszel method.

## 3. Results

### 3.1. Prevalence of *E. bieneusi* in Horses

Among the 412 horse fecal samples collected from four provincial‐level regions in China, 62 tested positive for *E. bieneusi*, corresponding to an overall infection prevalence of 15.0% (95% CI: 11.8%–18.7%) (Table 2). The highest prevalence was detected in Qiqihar, Heilongjiang Province (29.3%, 12/41; 95% CI: 16.1%–45.5%), followed by Chaoyang, Liaoning Province (15.9%, 11/69; 95% CI: 8.3%–26.7%), Kedong, Heilongjiang Province (15.2%, 7/46; 95% CI: 6.3%–28.9%), Harbin, Heilongjiang Province (14.1%, 20/142; 95% CI: 8.9%–21.0%), and Yakeshi, Inner Mongolia Autonomous Region (11.9%, 5/42; 95% CI: 4.0%–25.6%). The lowest prevalence was recorded in Wenzhou, Zhejiang Province (9.7%, 7/72; 95% CI: 4.0%–19.0%). Significant differences in *E. bieneusi* prevalence were observed across the six sampling locations (Fisher’s exact test, *p*  = 0.021). Using the Wenzhou group as the reference, crude PRs for the other regions were 1.22 (95% CI: 0.38–3.94) for Yakeshi, 1.45 (95% CI: 0.63–3.34) for Harbin, 1.57 (95% CI: 0.52–4.72) for Kedong, 1.64 (95% CI: 0.68–3.95) for Chaoyang, and 3.02 (95% CI: 1.27–7.19) for Qiqihar (Table 2).

**Table 2:** Prevalence of *E. bieneusi* infection in horses stratified by geographic region, host traits, and husbandry conditions.

Stratification category	Subgroup	No. tested	No. positive	Prevalence (%, 95% CI)	Crude PR (95% CI)	*p*‐Value
Geographic region	Zhejiang (Wenzhou)	72	7	9.7 (4.0–19.0)	1.00 (reference)	**0.021**
	Inner Mongolia (Yakeshi)	42	5	11.9 (4.0–25.6)	1.22 (0.38–3.94)	—
	Heilongjiang (Harbin)	142	20	14.1 (8.9–21.0)	1.45 (0.63–3.34)	—
	Heilongjiang (Kedong)	46	7	15.2 (6.3–28.9)	1.57 (0.52–4.72)	—
	Liaoning (Chaoyang)	69	11	15.9 (8.3–26.7)	1.64 (0.68–3.95)	—
	Heilongjiang (Qiqihar)	41	12	29.3 (16.1–45.5)	3.02 (1.27–7.19)	—
Host biological traits	>6 years	268	29	10.8 (7.4–15.1)	1.00 (reference)	**<0.001**
	1–6 years	115	19	16.5 (10.3–24.6)	1.53 (0.87–2.69)	—
	<1 year	29	14	48.3 (29.4–67.5)	4.47 (2.67–7.48)	—
	Female	292	32	11.0 (7.7–15.1)	1.00 (reference)	**0.001**
	Male^a^	120	30	25.0 (17.5–33.9)	2.28 (1.43–3.63)	—
	Non‐castrated	376	54	14.4 (11.0–18.3)	1.00 (reference)	0.248
	Castrated	36	8	22.2 (10.1–39.2)	1.54 (0.76–3.11)	—
Husbandry conditions	Equestrian club	147	17	11.6 (6.9–17.9)	1.00 (reference)	0.182
	Intensive farming	69	10	14.5 (7.2–25.0)	1.25 (0.57–2.74)	—
	Free‐range grazing	129	25	19.4 (13.0–27.2)	1.67 (0.92–3.04)	—
Live animal trading market		67	10	14.9 (7.5–25.7)	—	—
Overall		412	62	15.0 (11.8–18.7)	—	—

*Note:* Prevalence and 95% confidence intervals (CI) were calculated using the Wilson score method. Crude prevalence ratios (PR) and corresponding 95% CIs were estimated using the Mantel‐Haenszel method. *p*‐Values for overall inter‐group differences were calculated using Fisher’s exact test. No adjustment for multiple comparisons was performed; pairwise crude prevalence ratio values should be interpreted with caution genotype D (ON682482). Bold font highlights *p*‑values < 0.05 to mark statistically significant differences.

^a^The male group includes 36 geldings, which are further analyzed in the castration status subgroup to avoid sample overlap.

Age was significantly associated with *E. bieneusi* infection status (Fisher’s exact test, *p*  < 0.001). The highest prevalence was observed in foals aged <1 year (48.3%, 14/29; 95% CI: 29.4%–67.5%), followed by horses aged 1–6 years (16.5%, 19/115; 95% CI: 10.3%–24.6%) and adult horses aged >6 years (10.8%, 29/268; 95% CI: 7.4%–15.1%). Using the >6 years age group as the reference, the crude PR was 1.53 (95% CI: 0.87–2.69) for the 1–6 years group and 4.47 (95% CI: 2.67–7.48) for the <1 year group (Table 2). With respect to sex, male horses had a higher infection prevalence than female horses (25.0%, 30/120 vs. 11.0%, 32/292), and the difference was statistically significant (Fisher’s exact test, *p*  = 0.001). Compared with female horses (reference group), the crude *PR* for male horses was 2.28 (95% CI: 1.43–3.63). For castration status, prevalence was 22.2% (8/36; 95% CI: 10.1%–39.2%) in castrated horses and 14.4% (54/376; 95% CI: 11.0%–18.3%) in non‐castrated horses. No significant inter‐group difference was identified (Fisher’s exact test, *p*  = 0.248), with a crude PR of 1.54 (95% CI: 0.76–3.11) for castrated horses relative to the non‐castrated reference group (Table 2).

Among the three husbandry groups, free‐range grazing horses had the highest *E. bieneusi* prevalence (19.4%, 25/129; 95% CI: 13.0%–27.2%), followed by intensively farmed horses (14.5%, 10/69; 95% CI: 7.2%–25.0%) and horses kept in equestrian clubs (11.6%, 17/147; 95% CI: 6.9%–17.9%). No statistically significant difference in prevalence was observed across these three husbandry categories (Fisher’s exact test, *p*  = 0.182). Using the Equestrian Club group as the reference, crude PR values were 1.25 (95% CI: 0.57–2.74) for intensive farming and 1.67 (95% CI: 0.92–3.04) for free‐range grazing. Additionally, horses sampled from live animal trading markets had an infection prevalence of 14.9% (10/67; 95% CI: 7.5%–25.7%) (Table 2).

### 3.2. Genotypic Characterization of *E. bieneusi*

All 62 *E. bieneusi*‐positive isolates were successfully genotyped via ITS sequence analysis. Eighteen distinct genotypes were identified, comprising 11 known genotypes (CAF1, D, EbpC, GX3, HNR‐VI, horse1, Peru8, SHWR5, SYSWR18, SYSWR23, and Type IV) accounting for 55 isolates, plus 7 novel genotypes (HRBM1, KDM1, KDM2, QQHRM1, QQHRM2, QQHRM3, and WZM1), each represented by a single isolate (Table 3). Genotype D was the overwhelmingly predominant variant, constituting 58.1% (36/62) of all positive isolates. It also showed the widest geographic distribution, being detected in all four provincial‐level study regions. Genotype horse1 ranked second in prevalence at 14.5% (9/62) and was recovered from Zhejiang Province as well as Harbin and Kedong in Heilongjiang Province. Genotype EbpC (3.2%; 2/62) was identified in two horses from Qiqihar (Heilongjiang) and Chaoyang (Liaoning). The remaining eight known genotypes and all seven novel genotypes occurred as singletons with a sporadic distribution pattern. Confirmed zoonotic genotypes accounted for 67.7% of all positive isolates. The distributions of *E. bieneusi* genotypes stratified by host age, sex, and husbandry system are summarized in Table 3. Notably, all four rodent‐associated genotypes (HNR‐VI, SHWR5, SYSWR18, and SYSWR23) were detected exclusively in adult horses over 6 years of age and were mainly distributed in free‐range grazing and intensive farming systems with higher environmental exposure risk, providing preliminary clues for environmental spillover transmission from wild rodents to equines.

**Table 3:** Genotypic composition and diversity of *E. bieneusi* in horses across different subgroups.

Stratification category	Subgroup	Known genotypes (n)	Novel genotypes (n)
Geographic region	Harbin, Heilongjiang	**D (11)**; horse1 (7); **Peru8 (1)**	HRBM1 (1)
	Kedong, Heilongjiang	**D (3)**; horse1 (1); **Type IV (1)**	KDM1 (1); KDM2 (1)
	Qiqihar, Heilongjiang	**D (8)**; **EbpC (1)**	QQHRM1 (1); QQHRM2 (1); QQHRM3 (1)
	Yakeshi, Inner Mongolia	**D (3)**; **GX3 (1)**; SYSWR18 (1)	—
	Chaoyang, Liaoning	**D (6)**; **CAF1 (1)**; **EbpC (1)**; HNR‐VI (1); SHWR5 (1); SYSWR23 (1)	—
	Wenzhou, Zhejiang	**D (5)**; horse1 (1)	WZM1 (1)
Age group	<1 year	**D (13)**; **EbpC (1)**	—
	1–6 years	**D (15)**; horse1 (2)	KDM1 (1); KDM2 (1)
	>6 years	**D (8)**; horse1 (7); **CAF1 (1)**; **EbpC (1)**; **GX3 (1)**; HNR‐VI (1); **Peru8 (1)**; SHWR5 (1); SYSWR18 (1); SYSWR23 (1); **Type IV (1)**	HRBM1 (1); WZM1 (1); QQHRM1 (1); QQHRM2 (1); QQHRM3 (1)
Gender	Male	**D (16)**; horse1 (4); **CAF1 (1)**; HNR‐VI (1); **Peru8 (1)**; SYSWR23 (1); **Type IV (1)**; **EbpC (1)**	KDM1 (1); KDM2 (1); QQHRM3 (1)
	Female	**D (20)**; horse1 (5); **EbpC (1)**; **GX3 (1)**; SHWR5 (1); SYSWR18 (1)	HRBM1 (1); QQHRM1 (1); QQHRM2 (1)
Castration status	Castrated	**D (5)**; horse1 (1); **Peru8 (1)**	WZM1 (1)
	Non‐castrated	**D (31)**; horse1 (8); **EbpC (2)**; **CAF1 (1)**; **GX3 (1)**; HNR‐VI (1); SHWR5 (1); SYSWR18 (1); SYSWR23 (1); **Type IV (1)**	HRBM1 (1); KDM1 (1); KDM2 (1); QQHRM1 (1); QQHRM2 (1); QQHRM3 (1)
Husbandry system	Intensive farming	**D (6)**; **EbpC (1)**; HNR‐VI (1); SHWR5 (1); SYSWR23 (1)	—
	Free‐range grazing	**D (14)**; **CAF1 (1)**; **EbpC (1)**; **GX3 (1)**; horse1 (1); SYSWR18 (1); **Type IV (1)**	KDM1 (1); KDM2 (1); QQHRM1 (1); QQHRM2 (1); QQHRM3 (1)
	Equestrian club	**D (9)**; horse1 (6); **Peru8 (1)**	WZM1 (1)
Live animal trading market		**D (7)**; horse1 (2)	HRBM1 (1)
Total	—	**D (36)**; horse1 (9); **EbpC (2)**; **CAF1 (1); GX3 (1)**; HNR‐VI (1); **Peru8 (1)**; SHWR5 (1); SYSWR18 (1); SYSWR23 (1); **Type IV (1)**	HRBM1 (1); KDM1 (1); KDM2 (1); QQHRM1 (1); QQHRM2 (1); QQHRM3 (1); WZM1 (1)

*Note*: The genotypes in bold font indicate that they can infect both humans and animals.

### 3.3. Genetic Variation of *E. bieneusi*

Multiple sequence alignment revealed 30 polymorphic nucleotide sites across the aligned ITS sequences, with single nucleotide polymorphisms (SNPs) as the predominant variation type (Table 4). A unique 2‐bp deletion at positions 51–52 was detected exclusively in genotype WZM1. Homology searches against the GenBank database showed that all seven novel genotypes shared 97.94%–99.59% nucleotide identity with their closest reference sequences: HRBM1 differed from the globally prevalent genotype D (accession no. ON682482) by a single SNP at position 239 (G→C), with 99.59% identity; KDM1 harbored two nucleotide substitutions (106 A→G and 212 T→C) relative to genotype Encar2 (MG458707), with 99.17% identity; KDM2 showed one SNP at position 157 (C→T) compared with genotype SHH7 (PZ621295), with 99.59% identity; QQHRM1 carried two SNPs (83 T→A and 196 G→A) compared with genotype EbpC (MT557704), with 99.17% identity; QQHRM2 and QQHRM3 each carried three SNPs relative to their closest references, pigHN‐II (MF406105) and CHLJ‐E3 (OQ572343), respectively, both with 98.77% sequence identity; WZM1 was the only novel genotype with an insertion‐deletion (indel) variant, featuring a 2‐bp deletion at positions 51 and 52 plus two SNPs (135 A→G and 207 T→G) relative to genotype SHH3 (PZ621291). It had the lowest sequence identity (97.94%) among the novel genotypes (Table 5).

**Table 4:** Nucleotide variations at polymorphic sites of the ITS region among 18 *E. bieneusi* genotypes.

Genotypes	GenBank nos.	Nucleotide at position																															
		4	7	10	23	24	30	31	51	52	81	93	94	95	110	113	117	121	131	137	141	143	158	165	175	176	179	192	196	210	211	214	236
Known																																	
CAF1	PZ705416	G	T	G	C	G	T	G	G	T	C	T	A	G	C	T	G	A	G	C	T	A	T	G	T	A	T	G	A	G	G	G	G
D	PZ705408	G	T	G	C	G	T	G	G	T	C	C	A	G	C	C	T	A	G	C	T	A	T	G	T	A	T	G	A	G	G	G	G
EbpC	PZ705411	G	T	G	C	G	T	G	G	T	C	T	A	G	C	T	G	A	G	C	C	A	T	G	T	A	T	G	A	G	G	G	G
GX3	PZ705412	G	T	G	C	G	T	G	G	T	C	T	A	G	C	T	G	A	G	C	C	A	T	G	T	A	T	G	A	G	G	A	G
HNR‐VI	PZ705415	G	T	A	C	G	C	A	G	T	T	T	A	G	C	T	G	A	C	T	T	A	G	G	T	A	T	G	A	G	G	G	G
horse1	PZ705418	G	T	G	C	G	C	A	G	T	C	T	A	G	C	T	G	A	G	C	T	A	C	G	T	A	T	A	A	G	G	G	G
Peru8	PZ705409	G	T	G	C	G	T	G	G	T	C	C	A	G	C	C	G	A	G	C	T	A	T	G	T	A	T	G	A	G	G	G	G
SHWR5	PZ705417	G	T	G	C	G	T	G	G	T	C	C	A	G	C	T	T	A	G	C	T	A	T	G	T	A	T	G	A	G	G	G	G
SYSWR18	PZ705413	G	T	G	C	G	T	G	G	T	C	C	A	G	C	C	T	G	G	C	T	A	T	G	T	A	T	G	A	G	G	G	G
SYSWR23	PZ705414	G	T	G	C	G	T	G	G	T	C	C	G	G	C	C	T	A	G	C	T	A	T	G	T	A	T	G	A	G	G	G	G
TypeIV	PZ705410	G	T	G	C	G	T	G	G	T	C	T	A	G	C	C	G	A	G	C	T	A	T	G	T	A	T	G	A	G	G	G	G
Novel																																	
HRBM1	PZ705402	G	T	G	C	G	T	G	G	T	C	C	A	G	C	C	T	A	G	C	T	A	T	G	T	A	T	G	A	G	G	G	C
KDM1	PZ705403	T	T	T	C	G	T	A	G	T	C	T	A	G	T	C	G	A	G	C	T	A	T	G	T	A	T	G	A	G	G	G	G
KDM2	PZ705404	G	T	G	C	G	T	A	G	T	C	T	A	G	C	T	G	A	G	C	T	A	T	T	T	G	T	G	A	G	G	G	G
QQHRM1	PZ705405	G	C	G	C	G	T	G	G	T	C	T	A	G	C	T	G	A	G	C	C	A	T	G	T	A	A	G	A	G	G	G	G
QQHRM2	PZ705406	G	T	G	A	T	T	G	G	T	C	T	A	T	C	T	G	A	G	C	T	G	T	G	T	A	T	G	G	G	G	G	G
QQHRM3	PZ705407	G	T	G	C	G	T	G	G	T	C	T	A	G	C	C	G	A	G	C	C	A	T	G	T	A	A	G	A	A	A	G	G
WZM1	PZ705401	G	T	G	C	G	T	G	–	–	C	T	A	G	C	T	G	A	G	C	T	A	T	T	C	G	T	G	A	G	G	G	G

*Note*: The polymorphic sites are numbered relative to the reference genotype D (ON682482.1) of the internal transcribed spacer (ITS) region. The symbol “–” indicates nucleotide deletion. Genotype WZM1 row: positions 112 and 113 are marked with “–” to indicate the 2‐bp deletion.

**Table 5:** Nucleotide variations and sequence identity of the 7 novel *E. bieneusi* genotypes compared with their closest reference sequences.

Genotype	GenBank no.	Closest reference (accession no.)	Nucleotide variations	Nucleotide identity (%)
HRBM1	PZ705402	D (ON682482)	239 G→C	99.59
KDM1	PZ705403	Encar2 (MG458707)	106 A→G; 212 T→C	99.18
KDM2	PZ705404	SHH7 (PZ621295)	157 C→T	99.59
QQHRM1	PZ705405	EbpC (MT557704)	83 T→A; 196 G→A	99.18
QQHRM2	PZ705406	pigHN‐II (MF406105)	72 C→G; 141 A→T; 228 G→T	98.77
QQHRM3	PZ705407	CHLJ‐E3 (OQ572343)	91 G→A; 166 T→C; 204 C→G	98.77
WZM1	PZ705401	SHH3 (PZ621291)	112 delete; 113 delete; 135 A→G; 207 T→G	97.94

*Note:* Nucleotide positions are numbered relative to the corresponding reference genotype D (ON682482).

### 3.4. Phylogenetic Analysis of *E. bieneusi*

**Figure 2:**
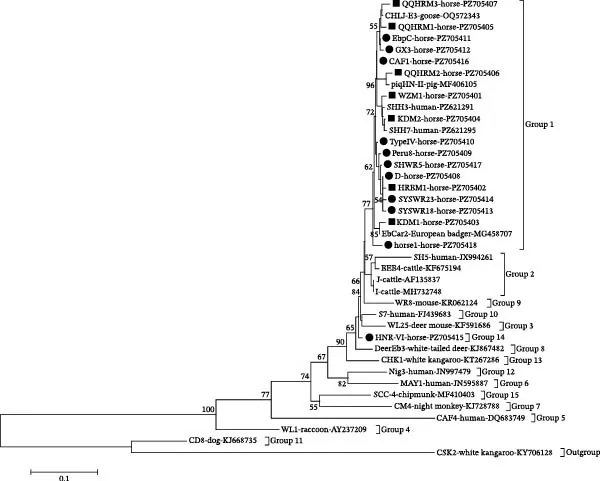
Phylogenetic relationships of *E. bieneusi* genotypes identified in this study based on ITS sequences. The neighbor‐joining tree was constructed using the Kimura 2‐parameter model with 1000 bootstrap replicates. Bootstrap values ≥ 50% are shown at the nodes. Groups are labeled on the right, and each terminal node indicates the genotype, host, and GenBank accession number of the corresponding sequence. The scale bar denotes a genetic distance of 0.1. Known and novel genotypes identified in this study are marked with solid circles and solid squares, respectively.

Phylogenetic analysis using the NJ method with the Kimura 2‐parameter model showed that all identified genotypes except HNR‐VI clustered within zoonotic Group 1. Each of the seven novel genotypes formed a distinct evolutionary branch adjacent to its closest reference genotype (Figure 2).

## 4. Discussion

Globally, *E. bieneusi* infection in equines has been documented in 12 studies across eight countries, with reported prevalence ranging from 1.3% to 30.9% (Table 1). The overall detection rate of 15.0% in our study is lower than the prevalence reported in the Czech Republic (17.5%) and Turkey (18.7%) but notably higher than rates from Iran (11.5%), Colombia (6.5%), Algeria (6.9%), the United States (8.3%), and the Republic of Korea (1.3%) (Table 1). Within China, existing epidemiological surveys of equine *E. bieneusi* show a wide prevalence range, from 2.4% in Shanghai to 30.9% in the Xinjiang Uygur Autonomous Region [12, 15]. The infection rate found in our study falls in the middle of the national range and is close to the prevalence reported in Qinghai Province (15.6%) [14].

At the subregional level, prevalence was closely associated with local husbandry patterns and environmental conditions. Qiqihar in Heilongjiang Province, where extensive free‐range grazing is the dominant rearing mode, exhibited the highest local carriage rate (29.3%), which is nearly consistent with the infection level (30.9%) observed in grazing equine herds in Xinjiang [12]. In contrast, Wenzhou in Zhejiang Province, where most sampled horses are kept in commercial equestrian clubs with comprehensive biosecurity measures, had the lowest regional prevalence (9.7%). This rate was nevertheless higher than the average infection rate (4.8%) previously reported from 17 equestrian clubs across 15 Chinese cities [15]. Across the three rearing systems, *E. bieneusi* prevalence decreased sequentially from free‐range grazing herds to intensively farmed horses and then to horses in professional equestrian clubs. This geographical and husbandry‐related prevalence gradient can be explained by multiple interrelated factors. First, northern provinces such as Heilongjiang, Inner Mongolia, and Liaoning harbour abundant domestic livestock (including pigs, cattle, goats, and sheep) and wild animal species such as deer and tigers [23–28]. These diverse hosts may serve as potential reservoir hosts for *E. bieneusi*, increasing environmental contamination and pathogen spread. Second, free‐ranging horses are continuously exposed to infective spores via contaminated forage, soil, and shared water sources, leading to more frequent and complex fecal–oral transmission [12]. Third, intensive horse farms generally have poorer environmental hygiene and less strict biosecurity management than commercial equestrian clubs. Individual stall housing, daily fecal removal, and regular enclosure disinfection practiced in equestrian clubs can effectively interrupt the fecal–oral transmission route of microsporidia [15]. As a distinct transmission setting, livestock trading markets had an infection rate of 14.9%. Although this rate was not markedly higher than that of fixed breeding facilities, the concentration of horses from geographically diverse origins at trading sites creates high‐risk nodes for cross‐regional pathogen spread, highlighting the need to include live animal trading venues in routine zoonotic parasite surveillance under the One Health framework.

Beyond these ecological and management drivers, cross‐study differences in *E. bieneusi* prevalence may also be influenced by multiple confounding factors, including host age, sex, sample size, and detection methodology [11]. Host age was identified as the strongest factor associated with *E. bieneusi* infection in our study: foals under 1 year of age had a 4.47‐fold higher positive rate than adult horses over 6 years old. This age‐dependent susceptibility has a clear immunological basis: equine intestinal mucosal barrier function and CD4^+^ T‐cell‐mediated IFN‐γ responses, which are critical for clearing intracellular microsporidia, do not fully mature until 6–12 months of age [29, 30]. Additional stressors such as weaning, abrupt dietary changes, and routine veterinary procedures further disrupt intestinal epithelial integrity and gut microbiota homeostasis, creating favorable conditions for spore attachment and intracellular proliferation. Our finding is consistent with two previous studies that also reported significantly higher *E. bieneusi* infection rates in young horses than in adults in Colombia and the Republic of Korea [9, 17]. However, inconsistent findings have also been reported. For example, studies in Algeria and the Czech Republic found no statistically significant difference in infection rates across age groups in horses. In contrast, a study of horses in northern China found that animals older than 12 months had a significantly higher *E. bieneusi* infection rate (31.1%) than horses younger than 6 months (3.4%) and those aged 6–12 months (3.8%) [11]. This discrepancy may reflect differences in the sample age structure and feeding management practices across study populations.

Although most published surveys of equine *E. bieneusi* have not identified host sex as a significant predictor of infection risk, our study found a higher prevalence in male than in female horses. This male‐biased infection pattern has also been reported in other mammalian hosts, including beef cattle, deer, sheep, goats, and wild fur‐bearing mammals [31–34]. Two interrelated mechanisms may account for this discrepancy. Behaviorally, male animals generally have larger home ranges and engage in more frequent environmental exploration, increasing their likelihood of ingesting infective spores from contaminated water, forage, and soil [35]. Immunologically, testosterone‐mediated immunomodulation may moderately reduce intestinal mucosal barrier function and cellular immune responses against intracellular microsporidian pathogens [36]. Nevertheless, a causal link between host sex and *E. bieneusi* susceptibility remains unproven, and further large‐scale epidemiological studies with balanced sex stratification are needed to confirm this association.

Globally, at least 59 distinct *E. bieneusi* genotypes have been documented in equine populations, with horse1, horse2, and genotype D being the most frequently reported. Twelve of these genotypes—BEB6, CHG19, CS‐4, CZ3, D, EbpA, EbpC, G, O, Peru8, Type IV, and WL15—are recognized as having zoonotic potential (Table 1) [8–19]. Genotype profiles and the proportion of zoonotic genotypes vary considerably across the study populations and regions. In general, the magnitude of zoonotic transmission risk is determined by two factors: overall infection prevalence and the relative abundance of zoonotic genotypes circulating in a given host population. For example, a survey in Xinjiang, China, found both a high infection rate and a high proportion of zoonotic genotypes, indicating a relatively high risk of horse‐to‐human transmission in this region [12]. In our study, 18 distinct genotypes were identified, comprising 11 known genotypes (D, horse1, Peru8, Type IV, EbpC, CAF1, GX3, HNR‐VI, SHWR5, SYSWR18, and SYSWR23) and 7 novel genotypes (HRBM1, KDM1, KDM2, QQHRM1, QQHRM2, QQHRM3, and WZM1). Genotype D was predominant, accounting for 58.1% of all isolates and detected at all six sampling sites, followed by genotype horse1, which represented 14.5% of isolates and was identified at three locations. Genotype D is the most commonly reported genotype in human *E. bieneusi* infections, with a wide global distribution. To date, this genotype has been detected in at least 91 host species, including humans, across more than 40 countries worldwide [4, 37]. In contrast, genotype horse 1, one of the most common equine‐adapted genotypes, has been detected in horses across seven countries (Table 1) but has not yet been documented in human clinical cases [4]. In China, genotype D has been detected in at least 23 animal species, as well as in environmental matrices such as various water bodies and mechanical vectors such as flies [38–40], reflecting a complex One Health transmission network linking humans, animals, and the environment. The high prevalence and wide geographic distribution of genotype D in equine populations observed in our study highlight the potential epidemiological role of horses in *E. bieneusi* dissemination. Conversely, genotype horse 1 appears to have relatively high host specificity for equids, being predominantly detected in horses and donkeys [11]. However, a recent study found that genotype horse1 can also infect rodents and birds, suggesting it may have a wider host range than previously thought [41]. Further epidemiological surveys and experimental studies are, therefore, needed to clarify its zoonotic potential.

Among the other known genotypes detected in our study, CAF1, EbpC, Peru8, and Type IV are all well‐characterized zoonotic genotypes with confirmed human pathogenicity [4]. Genotype CAF1 has been repeatedly reported in human populations in multiple African countries, including Cameroon, the Republic of the Congo, Madagascar, and Nigeria, since its first identification in humans in 2007 [42–44]. In China, this genotype has been commonly detected in pigs, deer, and cattle [45–47]. Although genotype CAF1 has not yet been reported in humans in China, its ability to infect humans and its cross‐species transmission capacity are well documented. Genotype EbpC was first identified in pigs in 1996 and subsequently reported in HIV‐positive patients in Peru in 2003 [48, 49]. To date, this genotype has been reported in at least 43 animal species across 15 countries, and it is also one of the most prevalent genotypes in children and domestic pig populations in Heilongjiang Province, China [4, 50, 51]. Genotype Peru 8 was originally isolated from human clinical cases in Peru and has since been identified in at least 15 host species across seven countries [4, 49]. In China, this genotype has been widely detected in humans, non‐human primates, rodents, and water bodies [39, 52, 53]. Genotype Type IV is frequently detected in both immunocompromised and immunocompetent human patients and infects a broad spectrum of domestic and wild mammals, with records covering at least 31 host species across 25 countries [4, 54]. In China, this genotype has also been widely identified in farm animals, pets, wildlife, and water environments, as well as in diarrheal patients, immunocompromised individuals, and other human populations. This suggests a complex transmission pattern with circulation among animals, the environment, and humans [4, 39, 55, 56]. Notably, although these zoonotic genotypes were only detected in one or two specimens each in our study, the potential of infected horses to serve as reservoirs for *E. bieneusi* transmission to humans and other animal species should not be underestimated.

The remaining five genotypes (GX3, HNR‐VI, SHWR5, SYSWR18, and SYSWR23) appear to have a relatively narrow host range, though this observation may be constrained by limited current surveillance data. Genotype GX3 was initially identified in HIV patients in Guangxi, China, and later detected in pigs [57, 58]. All other genotypes in this subset originate from wild rodents: SHWR5, SYSWR18, and SYSWR23 were previously recovered from wild *Rattus norvegicus* in Heilongjiang Province, while HNR‐VI has been reported in wild rats from multiple regions, including Hainan and Wenzhou [53, 59, 60]. Sympatric wild rodents commonly inhabit the peripheries of equine facilities and can introduce *E. bieneusi* spores into rearing environments by contaminating stored feed, drinking water, and bedding materials with infected feces, suggesting the potential for bidirectional cross‐species transmission between wild rodents and equine populations. Integrating systematic rodent surveillance and control measures into routine equine facility biosecurity protocols represents a practical One Health intervention to reduce environmental pathogen loading and lower cross‐species transmission risk. Continuous monitoring of *E. bieneusi* prevalence in environmental samples and sympatric wild rodent populations around infected equine facilities is warranted to further clarify the transmission dynamics of these genotypes.

Seven novel genotypes were identified in our study. Phylogenetic reconstruction and sequence homology analysis showed that all seven novel genotypes are genetically closely related to well‐characterized zoonotic genotypes within Group 1, with only 1–5 nucleotide differences across the entire ITS locus (Figure 2 and Table 5). Given that Group 1 is the major genogroup consistently linked to zoonotic transmission of *E. bieneusi* [3], these molecular features suggest that the novel genotypes may have zoonotic transmission capacity. Nevertheless, this inference remains preliminary and is based solely on ITS sequence similarity; direct evidence of human infection with these genotypes is still lacking. Further targeted epidemiological surveys of populations with frequent close contact with equids—including equestrian facility workers, pastoralists, and veterinary practitioners—are needed to confirm the actual public health risk posed by these novel genotypes.

The seven novel genotypes identified in our study further expand the global genotypic diversity of *E. bieneusi*. As summarized in a comprehensive review by Koehler et al. [4], the number of documented unique *E. bieneusi* ITS genotypes rose sharply from 100 in 2005 to 819 by June 2021, with China contributing a disproportionately large share of newly reported genotypes in recent years. For example, a survey of human populations in Hainan Province identified 18 novel genotypes, and a single study of brown rats in Heilongjiang Province detected as many as 61 new genotypes [59, 61]. This rapid expansion of known genotypes reflects the extremely high intraspecific genetic diversity of this pathogen. Because any nucleotide variation within the short ITS region (240–245 bp) is sufficient to define a novel genotype, many more genotypes are expected to be discovered as molecular surveillance expands to additional host species and geographic regions. In particular, studies of currently under‐sampled hosts—including wild rodents, invertebrate mechanical vectors, and free‐living wildlife—will be critical for building a more complete picture of *E. bieneusi* genotypic distribution and resolving its full transmission cycles.

### 4.1. Limitations and Future Research Directions

Several limitations of the present study should be acknowledged. First, as a cross‐sectional epidemiological survey, this study can only identify statistical associations between variables and cannot establish causal relationships between putative risk factors and *E. bieneusi* infection. Furthermore, all enrolled horses were clinically asymptomatic at sampling, so the association between *E. bieneusi* infection and equine gastrointestinal clinical signs could not be evaluated. Second, genotyping was performed using only the ITS region. This single‐marker approach provides limited phylogenetic resolution and is insufficient to fully resolve the population genetic structure of *E. bieneusi* and the direction of cross‐host transmission. Additionally, no human samples from horse‐exposed populations, environmental samples (water, soil, and feed), or sympatric wildlife samples were collected; thus, direct evidence of zoonotic transmission from horses to humans is lacking, and environmental transmission pathways cannot be comprehensively traced. Third, sampling was restricted to four provincial‐level regions in northern and eastern China, and major equine‐rearing areas such as the Xinjiang Uygur Autonomous Region, Sichuan Province, and Inner Mongolian grasslands were not covered. This may limit the generalizability of our findings to the national equine population. Additionally, sample sizes were unevenly distributed across age strata and husbandry system subgroups. The relatively small sample size of foals (<1 year) and intensive farming establishments may have reduced statistical power for inter‐group comparisons, and the non‐significant difference in prevalence across rearing modes (*p* = 0.182) may be partially attributable to insufficient sample sizes in some subgroups. Further targeted research is needed to address these gaps. Longitudinal cohort studies with clinical follow‐up are needed to clarify the causal effects of risk factors and the clinical outcomes of equine *E. bieneusi* infection. High‐resolution molecular typing approaches such as multilocus sequence typing (MLST), combined with synchronous sampling of exposed humans, environmental matrices, and sympatric wildlife, will help systematically elucidate transmission chains and quantify zoonotic risk. In addition, expanding epidemiological surveillance to major horse‐breeding regions across China will generate more representative prevalence and genotype data for the entire country.

## 5. Conclusions

This study provides baseline molecular epidemiological data of equine *E. bieneusi* infection across four provincial‐level regions in northern and eastern China. The overall infection prevalence was 15.0%, with obvious geographical heterogeneity across the sampling sites. Young age and male sex were associated with a higher infection risk, and free‐range grazing systems were linked to higher pathogen carriage rates. Among the 11 known genotypes identified, six (D, *n* = 36; EbpC, *n* = 2; CAF1, *n* = 1; GX3, *n* = 1; Peru8, *n* = 1; and Type IV, *n* = 1) have confirmed zoonotic potential, accounting for 67.7% of all genotyped animals. All genotypes identified here, including the seven novel genotypes, clustered within zoonotic Group 1, indicating their potential zoonotic relevance. These findings suggest that horses may act as an environmental reservoir for human pathogenic *E. bieneusi*. Meanwhile, the detection of rodent‐associated genotypes (HNR‐VI, SHWR5, SYSWR18, and SYSWR23) in horses suggests cross‐species transmission between sympatric wild rodents and equine populations. This study complements the limited baseline data on equine *E. bieneusi* in China and highlights the importance of integrated One Health surveillance covering equines, wildlife, the environment, and occupationally exposed populations. Targeted biosecurity measures, including routine screening, standardized disinfection, and health education for personnel, are recommended for equine facilities and live animal markets to reduce the zoonotic risk of *E. bieneusi*.

## Author Contributions

**Xin Peng**: writing – original draft, investigation, formal analysis, software. **Zhongkai Zhang:** writing – original draft, investigation, formal analysis, software. **Chuhan Yan**: writing – original draft, investigation, formal analysis, software. **Fanggu Zhu**: writing – original draft, investigation, formal analysis. **Weimeng Qin**: writing – original draft, sample collection, DNA extraction. **Wei Zhao**: writing – review and editing, writing – original draft, validation, software, resources, project administration, investigation, funding acquisition, formal analysis. **Jiangqiong Ke**: writing – review and editing, supervision, resources, conceptualization.

## Funding

This work was supported by the Basic Scientific Research Project of Wenzhou (Grant Y2023070).

## Disclosure

The funding sponsors had no role in study design, data collection and analysis, decision to publish, or preparation of the manuscript.

## Ethics Statement

The research protocol was reviewed and approved by the Research Ethics Committee of Wenzhou Medical University (Approval No. SCILL2021‐01). Prior to sample collection, verbal consent was obtained from animal owners whenever feasible. Only freshly voided fecal samples were collected, and no animal restraint or invasive procedures were performed during the sampling.

## Conflicts of Interest

The authors declare no conflicts of interest.

## Data Availability

The nucleotide sequences generated in this study have been deposited in the GenBank database under accession numbers: PZ705401–PZ705418.
